# MG53 permeates through blood-brain barrier to protect ischemic brain injury

**DOI:** 10.18632/oncotarget.7965

**Published:** 2016-03-08

**Authors:** Yonggang Yao, Bo Zhang, Hua Zhu, Haichang Li, Yu Han, Ken Chen, Zhen Wang, Jing Zeng, Yukai Liu, Xinquan Wang, Yu Li, Duofen He, Peihui Lin, Xinyu Zhou, Ki Ho Park, Zehua Bian, Zhishui Chen, Nianqiao Gong, Tao Tan, Jingsong Zhou, Meng Zhang, Jianjie Ma, Chunyu Zeng

**Affiliations:** ^1^ Department of Cardiology, Daping Hospital, The Third Military Medical University, Chongqing Institute of Cardiology, Chongqing, P.R. China; ^2^ Department of Surgery, Davis Heart and Lung Research Institute, The Ohio State University, Columbus, OH, USA; ^3^ Institute of Organ Transplantation, Huazhong University of Science and Technology - Tongji Medical College, Wuhan, China; ^4^ Department of Physiology, Kansas City University of Medicine & Bioscience, Kansas City, MO, USA; ^5^ Department of Neurology, Daping Hospital, The Third Military Medical University, Chongqing, P.R. China

**Keywords:** stroke, cell membrane repair, neuroprotection, TRIM72, tissue plasminogen activator

## Abstract

Ischemic injury to neurons represents the underlying cause of stroke to the brain. Our previous studies identified MG53 as an essential component of the cell membrane repair machinery. Here we show that the recombinant human (rh)MG53 protein facilitates repair of ischemia-reperfusion (IR) injury to the brain. MG53 rapidly moves to acute injury sites on neuronal cells to form a membrane repair patch. IR-induced brain injury increases permeability of the blood-brain-barrier, providing access of MG53 from blood circulation to target the injured brain tissues. Exogenous rhMG53 protein can protect cultured neurons against hypoxia/reoxygenation-induced damages. Transgenic mice with increased levels of MG53 in the bloodstream are resistant to IR-induced brain injury. Intravenous administration of rhMG53, either prior to or after ischemia, can effectively alleviate brain injuries in rats. rhMG53-mediated neuroprotection involves suppression of apoptotic neuronal cell death, as well as activation of the pro-survival RISK signaling pathway. Our data indicate a physiological function for MG53 in the brain and suggest that targeting membrane repair or RISK signaling may be an effective means to treat ischemic brain injury.

## INTRODUCTION

Stroke is a leading cause of mortality and long-term disability worldwide. The majority of strokes occur as ischemic brain injuries, resulting from the occlusion of cerebral arteries [1]. An ischemic stroke usually results in lower perfusion pressure and blood oxygen content, and the development of neurotoxic events, including calcium overload, glutamate excitotoxicity, oxidative stress, inflammation, and nitric oxide (NO) production, which lead to the death of neuronal cells. While timely restoration of ischemic blood flow is required to prevent tissue infarction, reperfusion with oxygenated blood causes exacerbation of brain injuries, otherwise known as ischemia-reperfusion (I/R) injury. Although previous studies show some agents have promising neuro-protective effects, including cinnamophilin, EGCG and oxyresveratrol, their protective effects have been shown to be indirect and limited [2, 3]. Thus, searching for direct and more effective treatments of brain I/R injury has proven elusive.

A recent series of studies from our group identified MG53 as an essential component of the cell membrane repair machinery [4–7]. MG53, also known as TRIM72, is a member of the tripartite motif-containing (TRIM) superfamily, which is constituted of proteins that possess a RING finger domain, one or two B-boxes and a coiled-coil (CC) domain [8]. MG53 acts as a sensor of oxidation to oligomerize and recruit intracellular vesicles to sites of membrane disruption for the formation of a repair patch. Genetic ablation of MG53 results in defective membrane repair and *mg53−/−* hearts are more susceptible to I/R induced injury [9]. While we have showed that upregulation of MG53 within a cell can enhance resistance to cellular disruption [4, 7, 9, 10], the therapeutic value of such findings cannot be easily accomplished since targeting *in vivo* MG53 expression would face technical challenges.

We found that acute injury to the cell membrane leads to the exposure of a signal that can be detected by MG53, allowing recombinant human MG53 (rhMG53) protein to repair membrane damage when provided in the extracellular space [11]. Using *in vivo* animal models, we showed that intravenous delivery of rhMG53 can repair membrane damage in skeletal muscle and alveolar cells, and ameliorate the pathology associated with muscular dystrophy [11] and acute lung injury [12]. Due to the blood-brain barrier, many neuro-protective agents, proven in *in vitro* studies, lose their effect in *in vivo* experiments. In this study, we sought to test whether rhMG53 can traverse the blood brain barrier to protect against ischemic stroke. We present evidence that increased level of MG53 in blood circulation can target injured brain tissue to facilitate repair of I/R injury in a transgenic mouse model. We also tested the therapeutic effect of rhMG53 in I/R-induced injury to the brain by using an *in vivo* rat model of acute brain injury. Our data suggest that rhMG53 can cross the blood brain barrier upon I/R injury to the brain, inhibit apoptosis, and activate the pro-survival RISK signaling in injured brain tissue. Together, our data suggest that targeting MG53-mediated tissue protection represents an effective means for treating ischemic stroke.

## RESULTS

### MG53 protects injury to neuron cells

**Figure 1:**
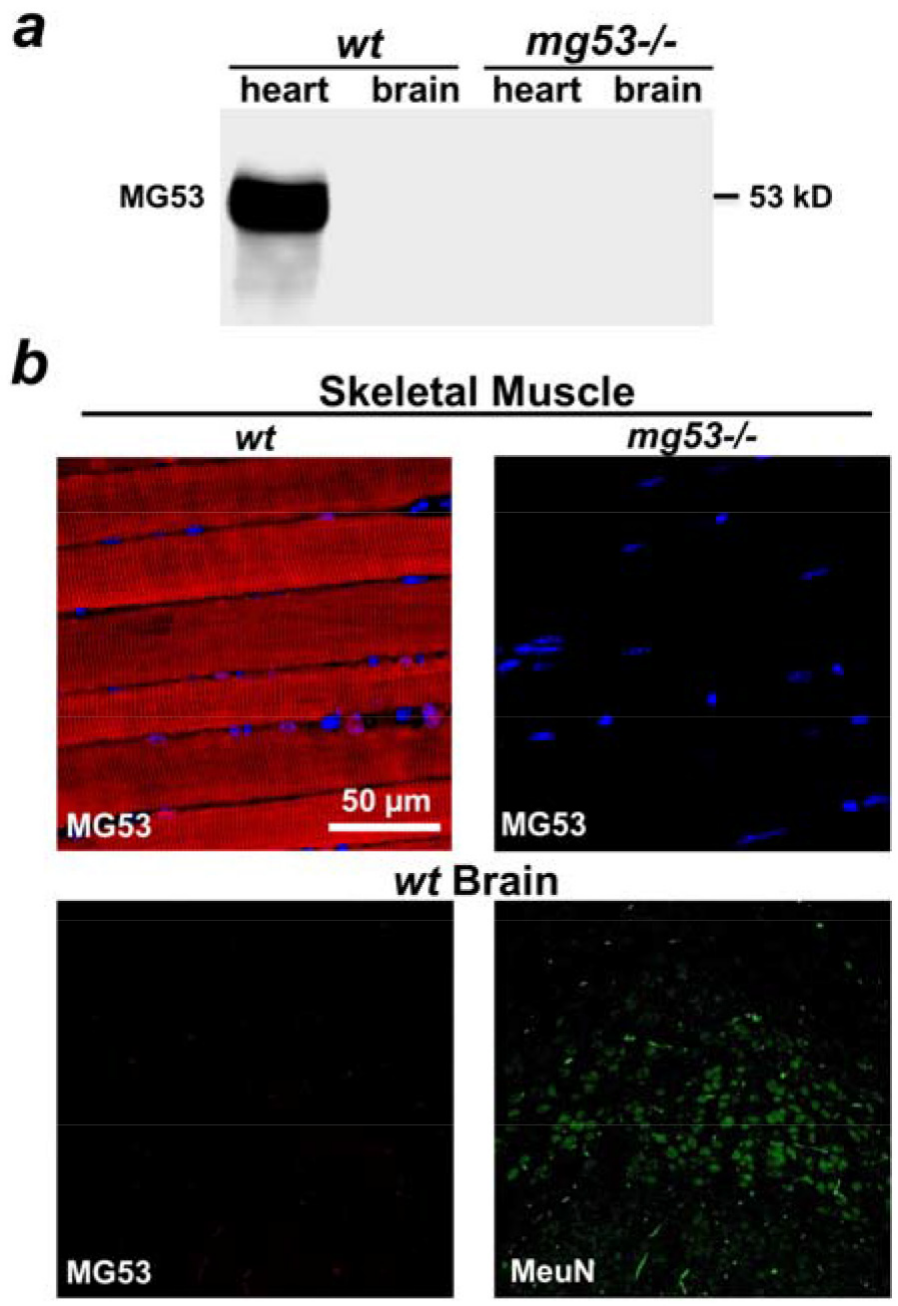
Lack of expression of MG53 in mouse brain tissue **a.** Western blotting showed that MG53 is present in the mouse heart, but not in the brain tissue. Tissues derived from *mg53−/−* mice were used as negative controls. **b.** Immunohistochemical staining revealed that MG53 is absent in brain tissue (lower left panel). Skeletal muscle tissue derived from *wt* mice (upper left panel) and *mg53−/−* mouse (upper right panel) were used as positive and negative controls respectively. NeuN staining was used to label neurons in brain slides (lower right panel).

**Figure 2:**
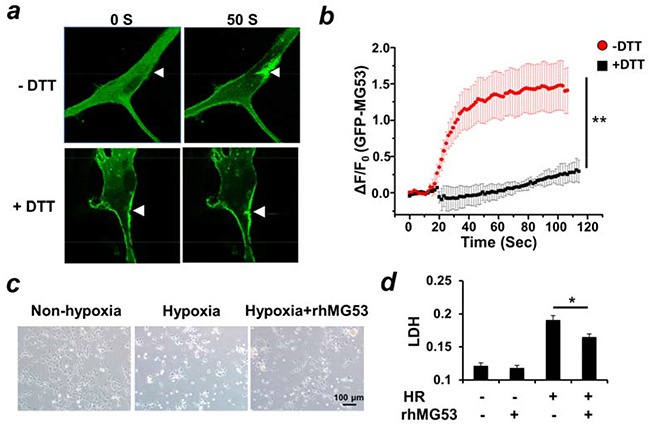
MG53 protects against injuries to neuron cells **a.** Microelectrode penetration of rat neuronal stem cells overexpressing GFP-MG53 led to nucleation of GFP-MG53 to injury site. The translocation of GFP-MG53 upon mechanical injury was inhibited by treatment of DTT (10 μM). **b.** Summary data for time-dependent accumulation of GFP-MG53 at the injury sites were plotted. Data are mean ± S.E. for n=8 cells from each group derived from 3 independent experiments. **P<0.01 +DTT vs. -DTT **c.** HT22 cells were treated with hypoxia for 18 hours and re-oxygenation for 6 hours. Treatment of rhMG53 preserved cell viability (c) and prevented LDH release as an indicator for plasma membrane injury **d,** *P<0.05 HR vs. HR+rhMG53, n=??

In mouse tissues, MG53 is predominantly expressed in skeletal and cardiac muscles [4, 13]. Immunoblotting showed no MG53 expression in the mouse brain (Figure 1a), and immunohistochemical staining of MG53 was negative in the mouse brain tissue (Figure 1b). Even though MG53 is highly enriched in striated muscles, it can accelerate the conserved cell membrane repair machinery in non-muscle cells to provide beneficial effects to targeted tissues [12, 14]. Cultured neuronal stem cells were infected with Ad-GFP-MG53 in order to investigate the extent of MG53's participation in the repair of membrane injury. As shown in Figure 2a, in response to injury caused by penetration of a micro-electrode into the plasma membrane, GFP-MG53 rapidly translocated toward the acute injury site. Nucleation of GFP-MG53 at the neuronal membrane injury sites was not observed when dithiothreitol (DTT) was present in the extracellular solution (Figure 2b). Such redox-dependent process of MG53-mediated cell membrane repair is similar to that observed in C2C12, HEK293, and other cell types [13, 15].

Previously we showed that rhMG53 protein can protect injury to various cell types when applied to the extracellular environment [11, 12, 14]. To mimic *in vivo* ischemia reperfusion (I/R) injury, we utilized an established hypoxia-reoxygenation (H/R) protocol that induces membrane injury to cultured HT22 neuron cells [16]. Leakage of lactate dehydrogenase (LDH) from the cell interior into the extracellular solution was used as an index of H/R-induced injury to HT22 cells. As shown in Figure 2c, the incubation of rhMG53 in the culture medium enhanced survival of HT22 cells following H/R treatment. Moreover, the application of rhMG53 significantly reduced the release of LDH from HT22 cells into the culture medium after H/R treatment (Figure 2d). This suggests that rhMG53 can protect neuron cells from I/R injury.

### Intravenous delivery of rhMG53 ameliorates I/R induced brain injury

**Figure 3:**
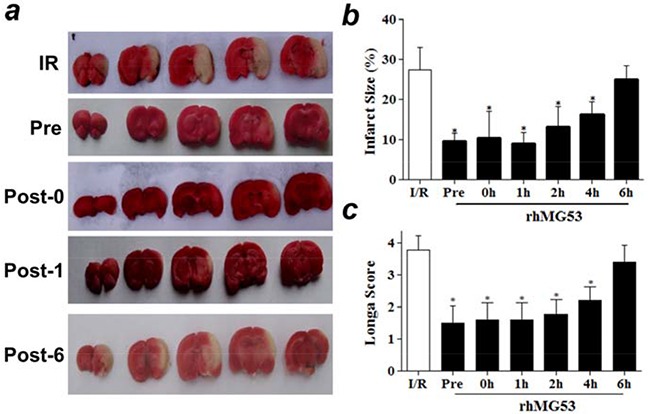
rhMG53 protects against I/R injury to rat brains **a.** Representative images of TTC staining of rat brain slides showed intravenous delivery of rhMG53 reduced infarct area as compared to saline treatment as controls. IR - rats receiving no treatment of rhMG53; Pre - rhMG53 administered prior to ischemia; Post-0, rhMG53 administered immediately after ischemia; Post-1, rhG53 administered 1 hour post reperfusion; and Post-6, rhMG53 administered 6 hours post reperfusion. **b.** Infarct sizes of different groups were quantified and summarized. rhMG53 treatment significantly reduced infarct area at different time points of administrations (pre, 0, 1h, 2h and 4h post reperfusion). The beneficial effect of rhMG53 was diminished when the animals were treated by rhMG53 6 h post reperfusion. *P<0.05 vs. I/R (n=7). **c.** Treatment of rhMG53 also led to significant improvement of behavior of the rats as demonstrated by Zea-Longa score. *P<0.05 vs. I/R (n=7 per group).

Based on the *in vitro* studies, we tested whether rhMG53 was effective at protecting against I/R-induced brain injuries in animal models. For I/R induced brain injury, Sprague-Dawley rats were subjected to 60 min of ischemia to the left carotid artery. A single dose of 3 mg rhMG53 protein/kg body weight was applied via left vena jugularis externa, either before or after the animals were subjected to I/R induced brain injury. Control animals received an equal volume of saline solution. As shown in Figure 3a, administration of rhMG53 prior to ischemia (*Pre*) could effectively reduce the area of infarction in the left brain. The protective effect of rhMG53 against I/R-induced brain injuries was also observed at different time points post reperfusion, with significant benefits seen up to 4 hours after (Figure 3b). Since administration of rhMG53 prior to and after ischemia can effectively protect I/R injury to the brain, one can potentially use rhMG53 for prevention as well as treatment of ischemic stroke. Our results also showed that MG53 loses its protective effect when administered 6 hours after reperfusion, thus establishing a time window for delivery of rhMG53 for effective treatment in rat models.

The neurologic behavior of rats was evaluated 24 hours after I/R injury to the brain using the Zea-Longa score method [17] by an investigator who was blind to the surgery operation and rhMG53 treatment paradigm. As shown in Figure 3c, a significant improvement in neurologic behavior was observed in rats that received rhMG53 treatment up to 4 hours post reperfusion of the brain injury; this is consistent with the infarct size measurement shown in Figure 3b. For visual demonstration of the behavior of the rats, we provide video recording of the animals that are subjected to I/R brain injury (receiving saline as control, Supplementary Movie S1), and I/R brain injury with rhMG53 administration (Supplementary Movie S2). One can see that rhMG53 treatment improves the animal behavior following brain injury.

**Figure 4:**
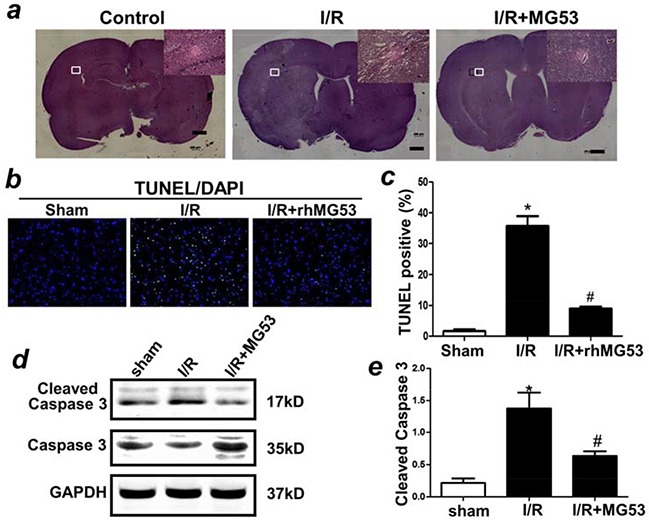
rhMG53 improves pathology and inhibits apoptosis of neural cells associated with I/R injury **a.** Histochemical analysis of brain slides indicated treatment of rhMG53 improved pathology associated with I/R injury. TUNELstaining **b, c.** and immunoblotting of caspase 3 **d, e.** indicated that treatment of rhMG53 inhibited apoptosis of neuron cells after I/R injury. *P<0.05 vs. sham (−rhMG53); #P<0.05 vs. I/R alone n=6.

rhMG53-mediated amelioration of brain anatomy was evident in H/E staining of the brain slices (Figure 4a). Clearly, I/R injury caused edema and damage in the injured side of the left brain, and rhMG53 treatment reduced the pathology. To determine the effect of rhMG53 on preventing apoptotic cell death in the brain, TUNEL measurement was conducted. As shown in Figure 4b and 4c, the percentage of I/R-induced TUNEL positive cells was significantly reduced following post treatment (after 1 h ischemia) of rhMG53. Brain tissues derived from the rats subjected to sham, I/R and IR/+rhMG53 were assayed for changes in caspase3 enzymatic activity as an indicator of apoptotic cell death (Figure 4d). Consistent with studies from other investigators [18–20], we found that I/R-induced brain injury led to activation of caspase3 activity; and rhMG53 treatment could suppress caspase3 activation (Figure 4d and 4e). Together, these results suggest that the reduction of apoptotic cell death contributes to MG53-mediated neuro-protection following I/R injury.

### Circulating MG53 can cross the blood-brain barrier to protect the mouse brain from I/R injury

**Figure 5:**
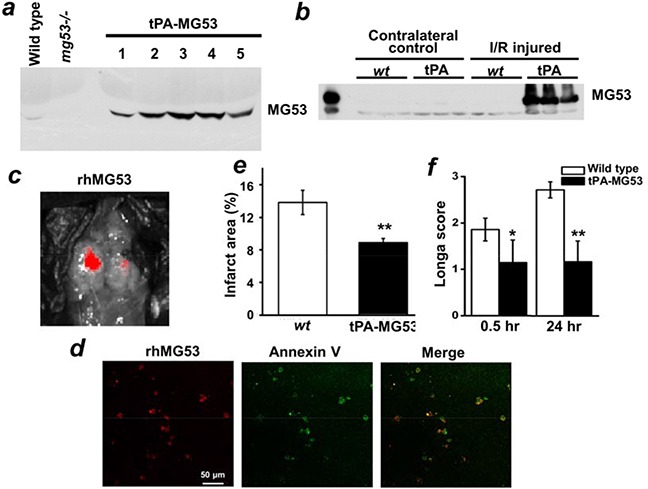
MG53 can cross the blood-brain-barrier to target to injured brain tissue **a.** Serum samples from *wt*, *mg53−/−* and tPA-MG53 mice were subjected to immunoblotting against MG53. The results showed tPA-MG53 expressed high level of MG53 in blood circulation. Western blotting **b.** showed that circulating MG53 targets to injury side of brain upon ischemic surgery. **c.** IVIS spectrum *in vivo* imaging showed rhodamine-labelled rhMG53 can target to the injured brain area, which was further confirmed by co-staining of rhodamine-labelled rhMG53 (red) and Annexin V-FITC (green) in brain slices **d.** When tPA-MG53 mice were subjected to I/R injury to the brains, they displayed resistance to I/R injury as compared to *wt* control, as evidenced by reduced infarct area **e.** and decreased Longa score **f.**

Previously we showed that MG53 present in the bloodstream plays a protective role against tissue injuries [11, 12, 14, 21]. To investigate the physiological function of circulating MG53 in neuro-protection, we constructed a transgene by adding a tissue plasminogen activator (tPA) leader sequence ahead of the *mg53* cDNA (tPA-MG53) to allow for secretion of MG53 into the bloodstream. The tPA-MG53 transgene was cloned behind a muscle-specific promoter for generation of the transgenic mouse model. As shown in Figure 5a, the tPA-MG53 transgenic mice displayed higher levels of MG53 in blood circulation (15.5±5.6 fold over the wild type level). With a limited cohort study (n=5 male and 5 female), we observed that tPA-MG53 mice have an increased life-span. Three out five male mice survived over 32 month, whereas all wild type control mice died before 30 months of age. Western blotting showed that the elevation of serum MG53 was maintained at the age of 32-month with the tPA-MG53 mice (not shown). Thus, this animal model provides us a unique tool for dissecting the paracrine function of MG53 in protection against brain injury.

With the tPA-MG53 mice, we detected no expression of transgenic MG53 in the brain tissue under healthy physiological conditions. Interestingly, I/R injury caused an abundant accumulation of MG53 in the brain tissue. While the contralateral right brain was negative for MG53 expression, the I/R-injured left brain was positive for MG53 expression (Figure 5b). This result raises the possibility that circulating MG53 can cross the blood-brain barrier (BBB) to target injured brain tissue.

To evaluate the BBB permeability of MG53, we labeled rhMG53 with a fluorescent marker (rhodamine). The rhodamine-labeled rhMG53 was intravenously administered to rats that were subjected to I/R induced injury to the left brain. As shown in Figure 5c, accumulation of rhMG53 in the left brain was observed, whereas the right brain only showed marginal labeling of rhodamine-rhMG53. Figure 5d show co-staining of rhodamine-rhMG53 and FITC-Annexin V in injured brain slices. The overlapping pattern of rhMG53 and Annexin V in the injured brain tissues is consistent with our previous study, demonstrating that the exposed phosphatidylserine serves as an anchoring mechanism for targeting of rhMG53 to the injury sites [11–14, 21]. Rats subjected to sham operation did not display any labeling of rhodamine-rhMG53 (not shown), indicating rhMG53 is impermeable to the BBB under healthy conditions.

We found that the tPA-MG53 mice were resistant to I/R-induced brain injury. The tPA-MG53 and their littermate control mice were subjected to 60 min of ischemia via occlusion of the left carotid artery. As shown in Figure 5e, TTC staining revealed a significantly lower development of brain injury in the tPA-MG53 mice, compared to their littermate controls. Moreover, Longa-score evaluation indicated significantly better neurological function in the tPA-MG53 mice 24 hours after I/R-induced brain injury (Figure 5f). Overall, these results suggest a neuro-protective role for circulating MG53 in the mouse stroke model.

### rhMG53 protects brain injury through activation of the RISK signaling pathway

**Figure 6:**
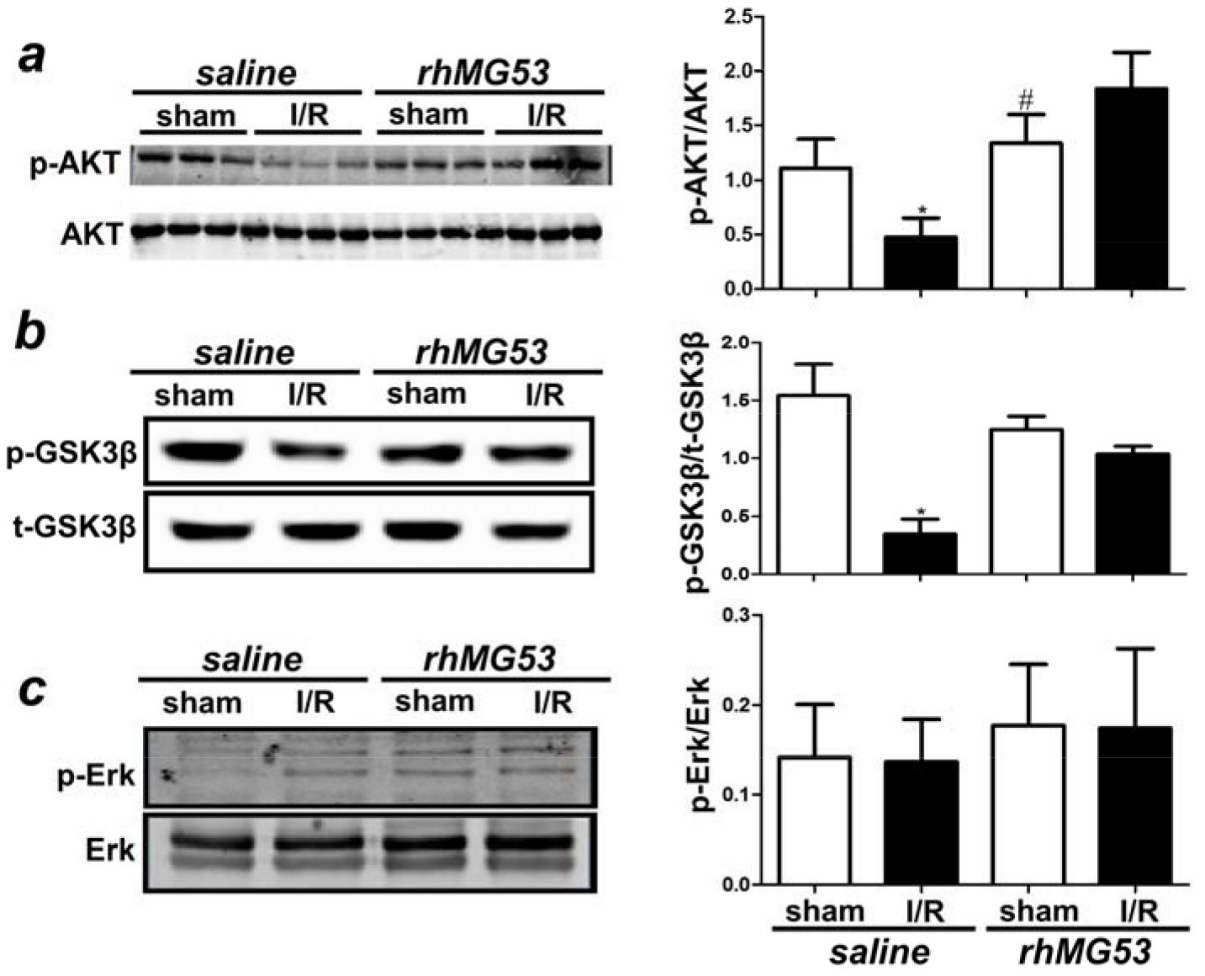
rhMG53 promotes pro-survival RISK pathway in response to I/R injury **a.** Western blotting of rat brain lysates showed that while I/R injury reduced phosphorylation of Akt (p-Akt) level, treatment of rhMG53 significantly promoted p-Akt in injured brains. **b.** Immunoblotting analysis also showed that treatment of rhMG53 significantly rescued decrease of phosphorylation of GSK3β (p-GSK3β) associated with I/R injury; whereas Erk pathway remained unchanged **c.** *P<0.05 vs. sham (−rhMG53); #P<0.05 vs. I/R alone, n=6.

**Figure 7:**
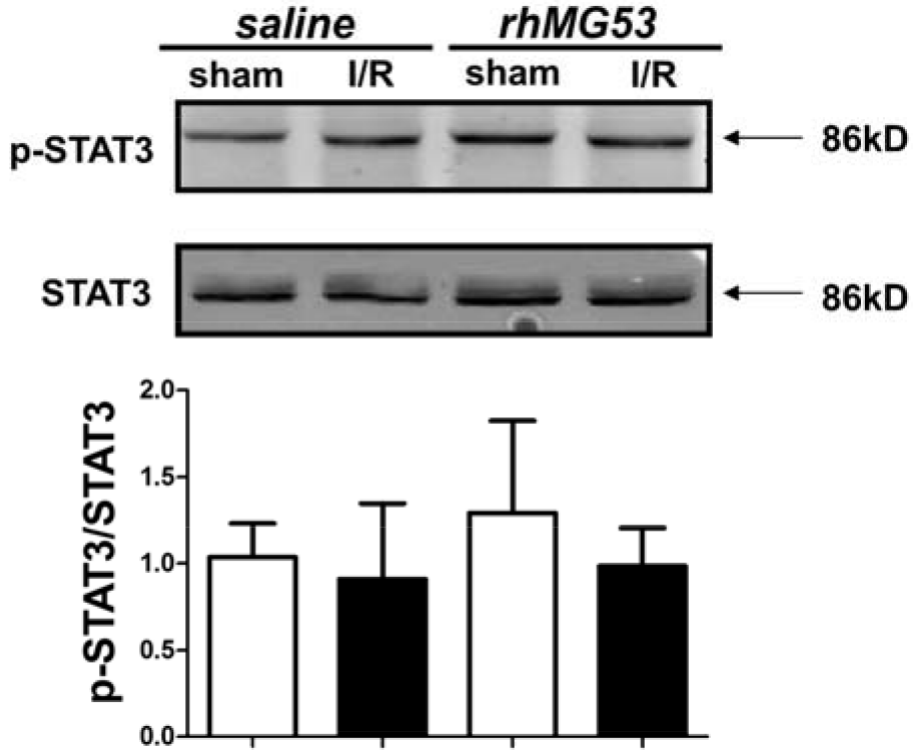
MG53 does not affect SAFE survival pathway I/R injury with or without MG53 treatment had no effect on phosphorylation of STAT3 in brain (P=NS, n=6).

Apoptosis is a major pathological outcome of brain tissue after I/R injury. Our data shown in Figure 4 indicate that, in addition to protecting against cell membrane injury, rhMG53 can reduce apoptotic cell death following I/R injury to the brain. Since RISK and SAFE are the major signaling components involved in apoptosis [22, 23] and our previous studies and others have demonstrated the involvement of RISK and SAFE pathways in cardioprotection [24–26], we tested if MG53 can modulate RISK and SAFE signaling in the brain. As shown in Figure 6a, I/R injury to the brain led to a reduction in AKT phosphorylation which can be restored by rhMG53 treatment. Similarly, GSK3β activation was suppressed after I/R injury and rhMG53 can restore GSK3β phosphorylation (Figure 6b). Interestingly, Erk signaling did not appear to be affected by I/R injury to the brain and rhMG53 did not affect its signaling (Figure 6c), which was also observed in the porcine model of myocardial infarction [21]. The SAFE pathway involves activation of STAT3 [27, 28]. We show in Figure 7 that STAT3 signaling is not affected by I/R injury to the brain, and rhMG53 did not affect STAT3 activation. Thus, SAFE is not involved in MG53-mediated neuro-protection. Overall, these results suggest that rhMG53 can activate the RISK survival pathway to elicit neuro-protection.

## DISCUSSION

In this study, we explored the therapeutic value for targeting MG53-mediated cell membrane repair to treat ischemic brain injuries. While MG53 in blood circulation is impermeable across the blood-brain-barrier (BBB) under healthy condition, ischemic injury to the brain increases the permeability of MG53 through the BBB, allowing MG53 to target the injury sites in the brain tissue. We provide evidence that sustained elevation of endogenous MG53 in the bloodstream or intravenous administration of exogenous rhMG53 protein both protect against ischemic brain injury. The neuro-protective effect of rhMG53 involves preservation of plasma membrane integrity as well as inhibition of apoptotic cell death in the infarct zone by activating RISK survival signaling pathway.

We developed a unique tPA-MG53 transgenic mouse model to demonstrate that secretion of MG53 from muscle tissues is linked to protection of remote organ injuries through circulation. Although MG53 is predominantly expressed in striated muscles, MG53 in blood circulation presents a broader physiological function in protecting tissues that do not express endogenous MG53. An important finding of our study is that MG53 in the bloodstream can cross BBB to target injured brain tissue, which supports the value for targeting MG53-mediated tissue repair in treatment of ischemic stroke. Our *in vivo* studies with the rat model formed a solid foundation by providing a time window as well as MG53 dosing information for our future clinical studies.

Current treatment of ischemic brain injury utilizes anti-thrombolytic reagents to dissolve the clot in the brain [29]. For examples, intravenous injection of tissue plasminogen activator (tPA) is widely used as a first treatment option for patients experiencing acute stroke [30]. However, tPA is known to cause tissue injuries, and can sometimes exacerbate the degree of brain damages associated with stroke. Thus, one therapeutic approach can involve a combination of rhMG53 and tPA to restore blood flow to the brain and to alleviate brain injuries during the reperfusion stages of stroke.

Delayed treatment is a major cause of death of the patients suffering ischemic stroke. In the present study, we found that rhMG53 can protect brain and preserve neuron functions 4 hours after injury, which provides a wider treatment window to perform either thrombolytic therapy or mechanical thrombectomy. In addition, the tissue protective function of rhMG53 might further work on the surgery during thrombectomy, allowing better recovery of patients from the invasive procedure bypassing the side effect of tPA anti-thrombotic activity. Since MG53 can protect multiple tissue injuries, one future research direction might focus on testing the potential protective effects of MG53 in protecting blood vessel to prevent excessive bleeding during surgery or ischemic stroke.

Resolving the signaling pathway that underlies control of secretion of MG53 from muscle tissues into the bloodstream is critical for understanding the physiological and pathophysiological function of MG53 in regenerative medicine. We need to identify the potential damage signals that can be recognized by circulating MG53 for targeting to the injured tissues [14], presumably through binding to a potential receptor present at the injury site. Since we consistently observed activation of RISK signaling pathway upon rhMG53 treatment in multiple organs, we expect that the potential receptor should also interact with RISK prosurvival pathway components. The extensive protective effect of rhMG53 on brain injury cannot be solely attributed to preservation of plasma membrane integrity. During the I/R process, the brain underwent metabolic transition, reactive-oxygen species (ROS) production which causes extensive mitochondria dysfunction. We show that rhMG53 can reduce apoptotic cell death in the brain. We recently demonstrated that metabolic stress can induce MG53 translocation to the mitochondria in skeletal muscle [14]. MG53 may serve to protect against I/R-induced injury to the brain cells. Future studies will be required to understand the mechanism underlying the MG53 interaction with mitochondria for increasing the cell survival under stress conditions.

Reperfusion Injury Salvage Kinase (RISK) and Survivor Activating Factor Enhancement (SAFE) pro-survival signaling pathways are two common defensive mechanisms that protect tissues against various injurious stresses, such as ischemia reperfusion injury [23]. RISK promotes survival of injured tissue via activation of PI3K/Akt and ERK signaling cascade [31], and SAFE involves activation of tumor necrosis factor (TNF) and STAT3 [27, 32]. While RISK pathway has been found to play protective roles in multiple tissues, the research about SAFE pathway is largely in cardiac protection, especially in pre- and post-conditioning mediated cardio-protection [33–36]. Given this background, it is not surprising that RISK but not SAFE pathway is involved in MG53-mediated protection against I/R induced brain injury. Thus, our study emphasized the role of RISK pro-survival pathway in brain injury, which can be a potential therapeutic target for treatment of traumatic brain injury.

Overall, our results established a novel application of rhMG53 in protection of ischemic stroke. In addition, rhMG53 might synergistically work with current therapies for a better and improved recovery of patients with ischemic stroke.

## MATERIALS AND METHODS

### Experimental animals

A transgenic mouse line with overexpression of secretory MG53 was generated by introducing a MG53 cDNA sequence led by a secretory signaling sequence (tPA) into the mouse genome. The founder mice were confirmed with elevated levels of MG53 in blood circulation by immunoblotting. Sprague-Dawley rats (200-240 g) were purchased from the Animal Center of Daping Hospital, maintained at temperature of 24±2°C with 45±10% relative humidity. The experimental protocol was approved by the Institutional Animal Care and Use Committee of The Third Military Medical University. All surgeries were performed under appropriate anesthesia, and all efforts were made to minimize animal suffering.

### Neuronal cell culture and membrane injury assay

Neuronal stem cells were isolated from subventricular zone of the brain derived from newborn pups of Fischer 344 rats according to the established protocol [37]. Cells were cultured in neuronal stem cell growth media - DMEM/F12 (Invitrogen, Carlsbad, CA, USA) containing B27 (1:50, Invitrogen), basic fibroblast growth factor (10 ng/ml, R&D, USA), epidermal growth factor (10 ng/ml, R&D, USA), and penicillin-streptomycin (100 IU/ml, Invitrogen) at a 37°C in a humidified 5% CO2 incubator. Cells were plated on matrigel (BD Bioscience) coated glass-bottom dishes (Bioptechs Inc.) and infected with adenovirus expressing Ad-GFP-MG53 [13] 1 day before cell wounding experiments. Live cell confocal imaging was acquired with a Radiance 2100 laser scanning confocal microscope with a 40X (1.3 NA) oil immersion objective to monitor intracellular trafficking of Ad-GFP-MG53 transiently expressed in neuronal stem cells. The cell growth medium was replaced with balanced buffer containing either 1 mM CaCl_2_ with or without 10 μM DTT before subjecting to membrane injury assay. For mechanical membrane damage, Ad-GFP-MG53 infected neuronal stem cells were penetrated by the tip of a micropipette attached to a micromanipulator, and the movements of GFP-MG53 were monitored following our published protocols [11, 13, 38].

### Hypoxia-reoxygenation treatment of cultured neuronal cells

HT22 cells were maintained at 37° in Dulbecco's modified Eagle's medium (DMEM) supplemented with 10% fetal bovine serum (FBS) and 1% penicillin/streptomycin [39]. Cells grown on 6-well plate were subjected to treatments of control (PBS) or rhMG53 (60 μg/ml) for 2 hours, then placed in an hypoxia chamber with 1% oxygen, 5% CO_2_ and balanced N_2_ at 37°C for 18 hours followed by reoxygenation for 6 hours. For membrane injury assay, 50 μl supernatant was transferred from the well to a new 96-well plate for lactate dehydrogenase (LDH) release assay using the LDH Cytotoxicity Detection Kit (Thermo Scientifics). Measurement of the release of LDH was used as an index of membrane injury [6, 11].

### Animal models of middle cerebral artery occlusion of brain injury

Rats were anesthetized with sodium pentobarbital (50 mg/kg, intraperitoneal) and turned to the supine position, fixed to the surgical table using surgical tapes. Transient focal cerebral ischemia was induced by middle cerebral artery occlusion as described in previous literatures [40, 41]. Briefly, the left carotid artery was exposed to separate the external carotid artery and the internal carotid artery, external carotid artery was occluded at the level the middle cerebral artery branches for 60 min, using a 4-0 monofilament nylon suture (Beijing Cinontech Co. Ltd, China). After 60 min, the occlusion was removed to allow for reperfusion for 24 hours. For sham-operated rats, all the arteries were exposed during the surgical period but the filament was not inserted into the middle cerebral artery. The body temperature of the rats was maintained at 37.5±0.5°C during the surgery using a heating pad and a thermometer. For the mouse ischemia-reperfusion brain injury model, the protocol is modified from previous studies [42]. Briefly, mice were anesthetized by isoflurane and maintained under anesthesia throughout the 1-hour occlusion period. The external carotid artery was incised and a blunt-tip 6-0 nylon monofilament was used to occlude the external carotid artery. After 60 minutes, the nylon thread and the common carotid artery ligature are removed. In the sham surgery group, the arteries are visualized but not disturbed.

### Immunoblotting

Isolated brain tissues were lysed in lysis buffer (50 mM Tris-HCl, pH 7.4, 150 mM NaCl, 2 mM EGTA, 1 mM EDTA, 1% Triton X-100, 1mM phenylmethylsulphonyl fluoride and 10 mg/ml each leupeptin and aprotinin). After centrifugation at 20,000g for 30 minutes, the supernatants were collected and protein concentrations measured. The brain tissue homogenates (50 mg protein) were separated by 10% SDS–PAGE and transferred onto polyvinylidene fluoride membranes (Bio-Rad). The blots were washed with Tris-buffered saline with 0.05% Tween-20 (TBST), blocked with 5% milk in TBST buffer for 1 h and incubated with rabbit monoclonal anti-MG53, caspase 3, Akt, ERK, STAT2 antibodies were purchased from Cell Signaling, and the appropriate secondary antibody coupled to horseradish peroxidase (Sigma Co., 1:5,000 dilution). Bands were visualized with an ECL kit (Pierce), and quantified by Image Lab image acquisition and analysis software (Bio-Rad).

### Isolation and administration of recombinant human MG53 protein

Purification of rhMG53 protein has been described previously [11]. rhMG53 was lyophilized and stored at 4°C, as dry powder in a desiccator. For intravenous injection of rhMG53, the protein was diluted in 0.9% sterile saline, filtered through a 0.2-mm filter and injected via the tail vein. Rats were randomly divided into three groups: sham, I/R and I/R injury with MG53 treatment group. For the I/R treatment group, rhMG53 was given at different time points: 30 minutes before ischemia, immediately after ischemia, and different time points (1-6 hour) after reperfusion. The animal surgery was performed by one person, rhMG53 was administered by another person (Jing Zeng); both treatments were in a blinded manner to the third person who will conduct physiological and pathophysiological evaluations. rhMG53 was given via left vena jugularis externa through catheterization with polyethylene tubing (outer diameter ¼ 0.965 mm; inner diameter ¼ 0.58 mm). The dosage of rhMG53 was 3 mg/kg as previously described [11]. The sham operation and I/R groups were treated with the same volume of saline as control.

### Neurologic Zea-Longa score

The neurologic behavior of mice and rats was scored at 24 hours after reperfusion according to the Zea-Longa score by an investigator who was unaware of animal grouping. The neurologic findings were scored on a five-point scale: (1) 0 score, no neurologic deficit; (2) 1 score, failure to extend left forepaw fully-a mild focal neurologic deficit; (3) 2 score, circling to the left-a moderate focal neurologic deficit; (4) 3 score, falling to the left-a severe focal deficit; and (5) 4 score, did not walk spontaneously and had a depressed level of consciousness.

### Infarct volume measurement

For measurements of infarct volume, mice and rats were sacrificed by overdose of pentobarbital. Brains were rapidly removed and immediately frozen at −20°C for 20 μminutes to keep the morphology intact during slicing. Brains were sliced into five serial 2°mm coronal sections and incubated in a 1% 2,3,5-triphenyltetrazolium chloride (TTC; Sigma Co, USA) for 20 minutes at 37°C and turn over the slice gently every 5 minutes, fixed in 4% paraformaldehyde in phosphate buffer at 4°C. The white tissue indicated the infarct part, whereas the red meant normal. The extent of ischemic infarction was traced and the integrated volume was calculated using Image J software (NIH).

### Histological analysis

The brain tissue from rats subjected to I/R injuries were washed with ice-cold oxygenated saline to remove blood and then fixed in 4% phosphate-buffered paraformaldehyde for 2 days at 4°C before processing for paraffin embedding. Brain slices were sectioned (4mm), mounted on slides, deparaffinized and rehydrated by incubated successively in xylene, 100% ethanol, 95% ethanol, 75% ethanol and PBS. Sections were stained with hematoxylin and eosin using standard procedures and scored according to a four-point scale 25 by an experienced histologist from the Department of Pathology at Daping Hospital under blinded conditions.

### TUNEL staining and caspase-3 protein quantification

To evaluate the effect of rhMG53 on neuronal survival under ischemic conditions, TUNEL staining and caspase-3 enzymatic assay were used to detect apoptotic cell death and total apoptosis of brain tissues. TUNEL staining was performed with a kit (Roche, Mannheim, Germany). The TUNEL-positive nuclei with chromatin condensation and fragmented nuclei were considered as probable apoptotic cells. The quantification of caspase-3 protein was detected by immunoblotting.

### Small animal *in vivo* imaging

rhMG53 was conjugated with rhodamine by a dye labeling kit (G-Biosciences, St. Louis) and applied to rats immediately after ischemia, together with Annexin V-FITC (Biovision Inc.) administration in the left vena jugularis externa. After 1 hour, fluorescent signals in the rat brain were imaged using the IVIS spectrum pre-clinical *in-vivo* imaging system (PerkinElmer, Waltham, MA). All *in-vivo* images were analyzed using Living Image In-Vivo Imaging Software. After imaging, the brain tissue was removed immediately to cut into frozen sections. Immunofluorescence confocal images were acquired (Olympus AX70 laser confocal microscopy) at excitation wavelengths of 350 and 507 nm, emission was detected at 450 and 529 nm to observe the confocal images of rhodamine-labelled rhMG53 and Annexin V-FITC in hippocampal areas.

### Statistical analysis

The data are expressed as mean ± SD. Comparison within groups was made by Student's t-test when comparing two groups and by analysis of variance for repeated measures. Comparison among groups was performed with 1-way ANOVA followed by Holm–Sidak test. Differences were considered significant at P <0.05.

## SUPPLEMENTARY MOVIES


