# Supplementary figures and images for: MG53 permeates through blood-brain barrier to protect ischemic brain injury
Source: Oncotarget. 2016 Mar 8;7(16):22474–85. doi: 10.18632/oncotarget.7965 (PMC5008374; doi:10.18632/oncotarget.7965)

## SUPPLEMENTARY MOVIES

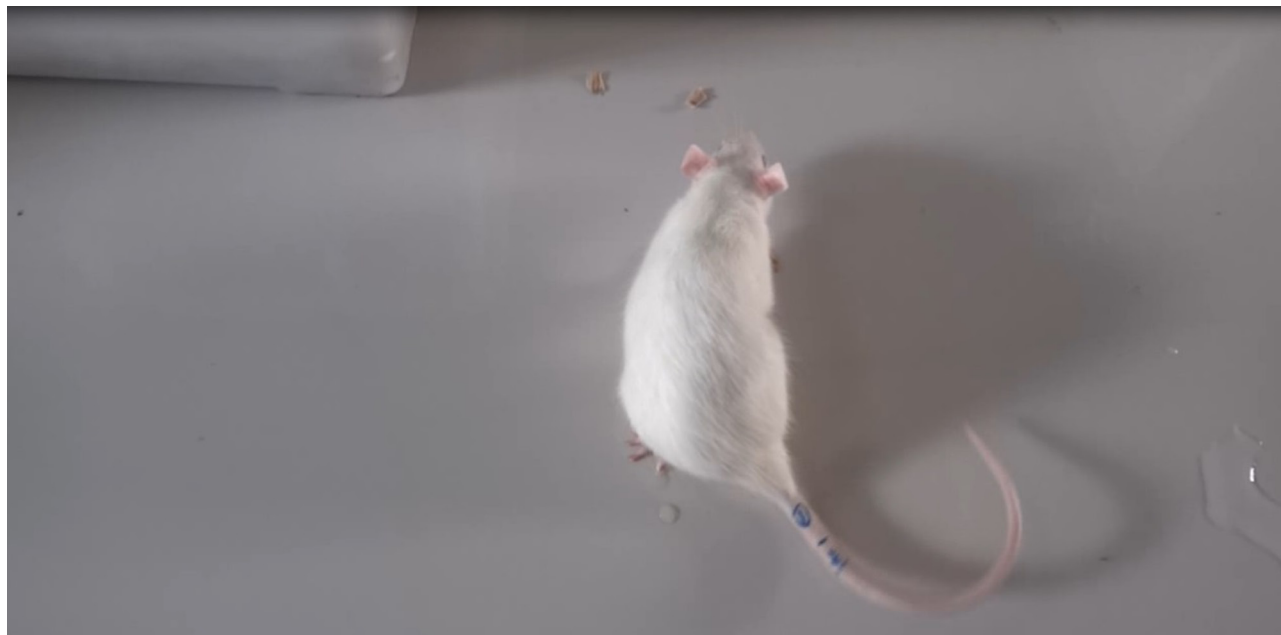

Supplementary Movie S1

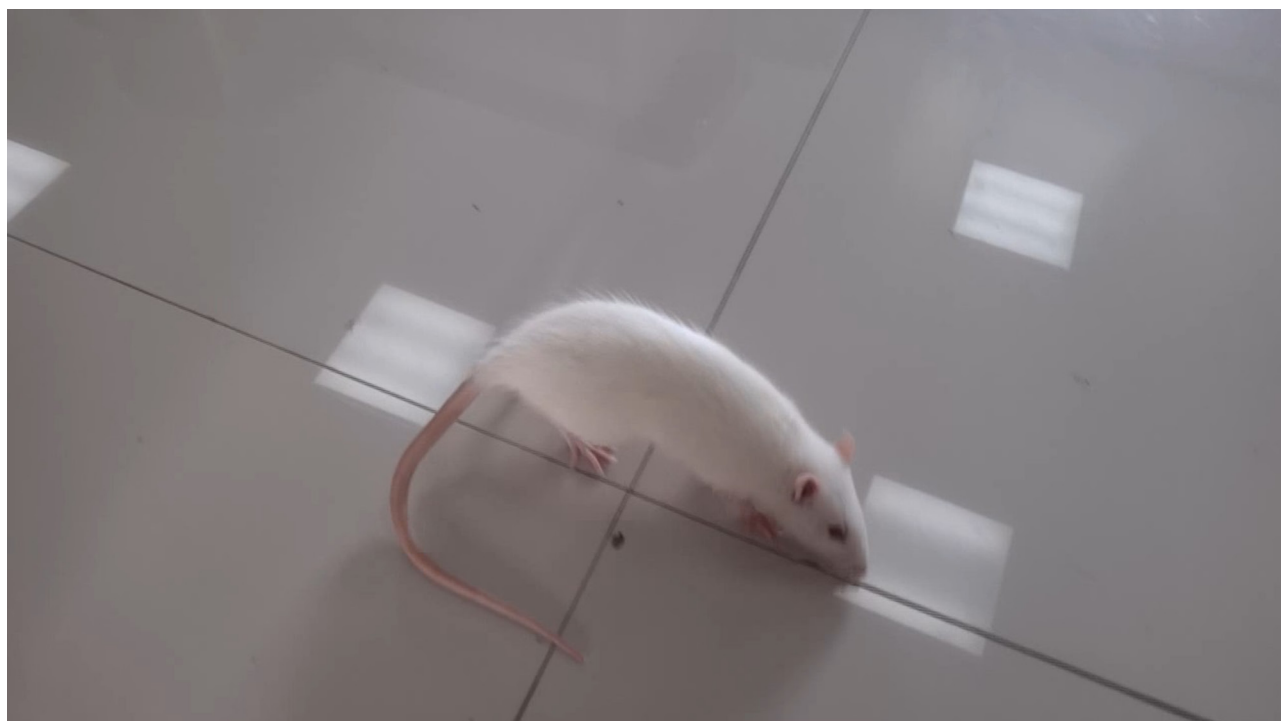

Supplementary Movie S2

Supplement: Supplementary file 1 [file oncotarget-07-22474-s001.pdf]
